# Heparin-free after 3000 IU heparin loaded in veno-venous ECMO supported acute respiratory failure patients with hemorrhage risk: a novel anti-coagulation strategy

**DOI:** 10.1186/s12959-022-00396-w

**Published:** 2022-06-27

**Authors:** Yang-Chao Zhao, Xi Zhao, Guo-Wei Fu, Ming-Jun Huang, Xing-Xing Li, Qian-Qian Sun, Ya-Bai Kan, Jun Li, Shi-Lei Wang, Wen-Tao Ma, Qin-Fu Xu, Qi-Long Liu, Hong-Bin Li

**Affiliations:** 1 Department of Extracorporeal Life Support Center, Department of Cardiac Surgery, The First Affiliated Hospital of Zhengzhou University, JianShe Road 1, Zhengzhou, 450052 Henan China; 2 Department of Cardiology, Henan Key Laboratory of Hereditary Cardiovascular Diseases, The First Affiliated Hospital of Zhengzhou University, Cardiovascular Center, Zhengzhou, 450052 Henan China; 3 Department of Respiration, The First Affiliated Hospital of Zhengzhou University, Zhengzhou, 450052 Henan China; 4 Department of Surgery ICU, The First Affiliated Hospital of Zhengzhou University, Zhengzhou, 450052 Henan China

**Keywords:** Anticoagulation, Extracorporeal membrane oxygenation, Complications, Hemorrhage, Mortality

## Abstract

**Background:**

The anti-coagulation protocol of patients with hemorrhage risk primary disease who need extracorporeal membrane oxygenation (ECMO) supported is controversial. This study evaluated the feasibility of a new anti-coagulation strategy, that is heparin-free after 3000 IU heparin loaded in veno-venous ECMO (VV ECMO) supported acute respiratory failure patients with hemorrhage risk.

**Methods:**

A retrospective study was performed in a series of hemorrhage risk patients supported with VV ECMO at the First Affiliated Hospital of Zhengzhou University, between June 2012 to Sept 2020. A total of 70 patients received a low heparin bolus of 3000 units for cannulation but without subsequent, ongoing heparin administration. Patients were divided into survival (*n* = 25) and non-survival group (*n* = 45). Data of coagulation, hemolysis and membrane lung function were calculated and analyzed. The complications of patients were recorded. Finally, the binary Logistic regression was conducted.

**Results:**

The longest heparin-free time was 216 h, and the mean heparin-free time was 102 h. Compared with survivors, the non-survivors were showed higher baseline SOFA score and lower platelet counts in 0.5 h, 24 h, 48 h and 96 h after ECMO applied. However, there was no significant differences between survivors and non-survivors in ACT, APTT, INR, D-dimer, fibrinogen, LDH, blood flow rate, Δp and P_post-ML_O_2_ (all *p* < 0.05) of all different time point. Moreover, only the baseline SOFA score was significantly associated with mortality (*p* < 0.001, OR(95%CI): 2.754 (1.486–5.103)) while the baseline levels of ACT, APTT, INR, platelet, D-dimer, fibrinogen and LDH have no association with mortality. The percentage of thrombosis complications was 54.3% (38/70) including 3 oxygenator changed but there was no significant difference of complications in survival and non-survival groups (*p* > 0.05).

**Conclusions:**

The anticoagulation protocol that no heparin after a 3000 units heparin bolus in VV ECMO supported acute respiratory failure patients with hemorrhage risk is feasible.

## Introduction

Venovenous extracorporeal membrane oxygenation (VV ECMO) implementation is a resuscitation strategy for patients with severe reversible refractory respiratory failure [1], and its use is increasing as its benefits are recognized. However, complications, particularly bleeding and thromboembolic events, are potentially life-threatening during ECMO support [2, 3]. Activated partial thromboplastin time (APTT) and activated clotting time (ACT) are commonly used clinically to adjust the dosage of unfractionated heparin (UFH) to reduce the incidence of complications. Current guidelines recommend an ACT-guided approach, aiming for a 1.5-fold increase from normal [4], rather than maintaining ACT within 120–180 s and APTT between 40–80 s [5]. Despite strict clinical adherence to ACT-guided heparin anticoagulation protocols, the incidence of bleeding complications exceeds 50% [3]. There is a great need for alternative anticoagulation strategies to reduce the risk of bleeding and thromboembolism.

Patients with acute respiratory failure following multiple trauma and surgery and those with acute respiratory failure with a history of gastrointestinal or airway bleeding are at risk of severe bleeding or re-hemorrhage. The incidence of bleeding complications during VV ECMO support is significantly higher in patients at high risk of bleeding than in other patients. Anticoagulation strategies therefore need to be developed for patients at high bleeding risk. Previous case reports have found heparin-free to be generally safe in critical ill patients, but conclusions are limited [6, 7].

Our study is the first to apply a heparin-free regimen after loading 3000 units of heparin to patients at risk of bleeding supported by VV ECMO, and we hypothesized that the new strategy is feasible.

## Materials and methods

### Study design and patients

**Fig. 1:**
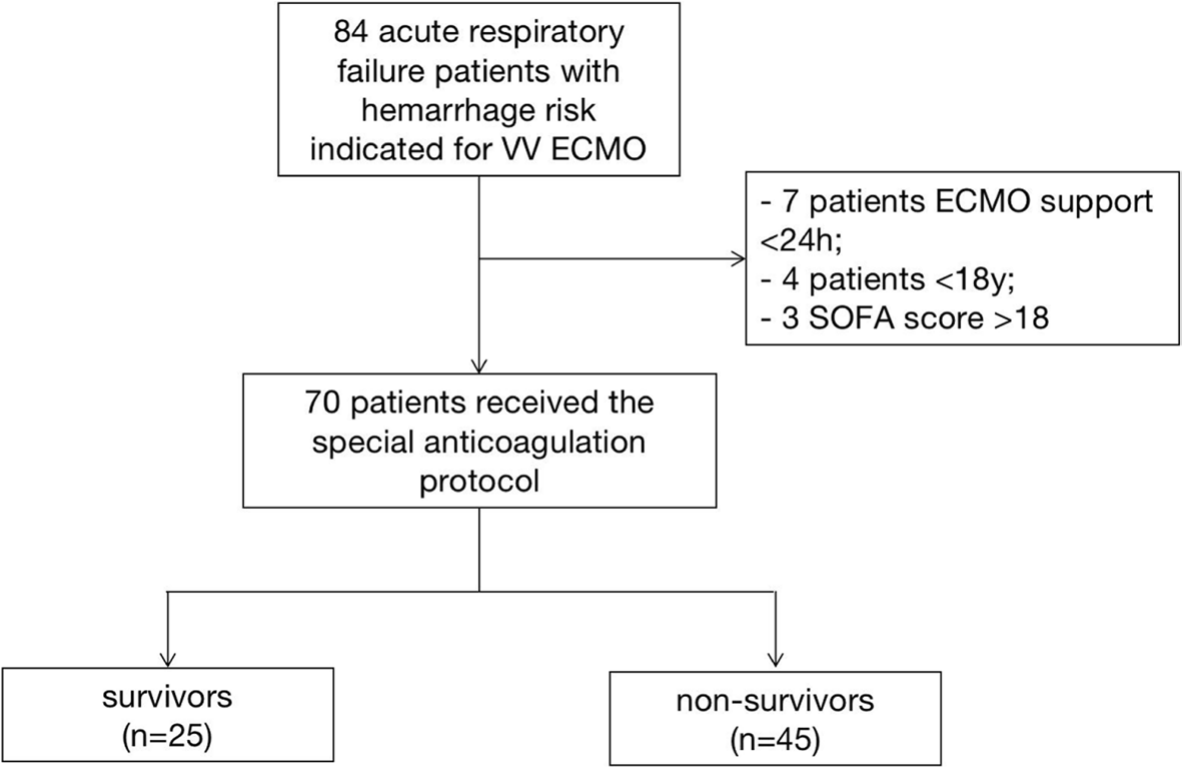
The flow chart

The present study retrospectively enrolled 70 severe acute respiratory failure patients receiving VV ECMO with hemorrhage risk in the First Affiliated Hospital of Zhengzhou University immediately from June 2012 to Sept 2020. 70 enrolled patients were divided into survival group (*n* = 25) and non-survival group (*n *= 45) (Fig. 1). In this study, the low heparin protocol was defined as 3000 units of heparin intravenously at the time of ECMO initiation and no ongoing heparin administration, as long as Δp were kept below 30 mmHg and P_post-ML_O_2_ were over 200 mmHg. ECMO system used was BE-PLS 2050 (Maquet, Rastatt, Germany). The cannulation of femoral vein and jugular vein were 17–25 French (Fr) cannulas.

Inclusion criteria were: 1) VV-ECMO support longer than 24 h; 2) potentially hemorrhage risk (trauma, history of gastrointestinal or airway hemorrhage, and after surgery). The exclusion criteria were as follows: 1) aged<18 years old; 2) pregnancy; 3) irreversible multiple organ failure; 4) uncontrolled metastatic malignancy; 5) severe craniocerebral injury; 6) active bleeding; 7) preexisting indication for therapeutic anticoagulation; 8) contraindication to heparin; 9) missing informed consent.

ACT was measured at the initiation(0 h), 0.5 h, 1 h, 2hs, 4hs, 8hs, 16hs, 24hs, 48hs, 72hs, 96hs, 120hs, 144hs, 168hs, 192hs and 216hs after ECMO running. APTT, international normalized ratio (INR), D-dimer and Fibrinogen were measured at 0 h, 0.5 h, 8hs, 16hs, 24hs, 48hs, 72hs, 96hs, 120hs, 144hs, 168hs, 192hs and 216hs. The levels of platelet and lactate dehydrogenase (LDH) were measured at 0 h, 0.5 h, 24hs, 48hs, 72hs, 96hs, 120hs, 144hs, 168hs, 192hs and 216hs. The blood flow rate, the Δp and P_post-ML_O_2_ of membrane lung were recorded at 0.5 h, 1 h, 2hs, 4hs, 8hs, 16hs, 24hs, 48hs, 72hs, 96hs, 120hs, 144hs, 168hs, 192hs and 216hs. The routine Doppler of heart and thrombosis (inferior vena cava, deep and superficial arteries and veins of both lower extremities, jugular arteries and veins, as well as inside and outside the cannula) was repeated once a day in the patients during the ECMO assisted. The time of ECMO assisted, mechanical ventilation and intensive care unit (ICU) stay were collected. The severity of the illness was assessed based on the sepsis-related organ failure assessment (SOFA) score before ECMO initiation. Finally, the complications were recorded.

### Endpoints

The primary endpoint was 30-day ICU mortality. Secondary endpoints were symptomatic thromboembolic events, ECMO oxygenator change and severe bleeding complications. The severe bleeding was defined as need for intervention or ≥ 10 red blood cell transfusions [8].

Routine threshold for platelet transfusion was < 50 × 10^9^/L but could be individualized depending on the clinical situations. Fibrinogen or cryoprecipitate could be infused when the level of blood fibrinogen was less than 2 g/L. When the level of D-dimer was too high, tranexamic acid could be used in combination with the condition.

Oxygenator change was considered in the following situations: 1) decreasing P_post-ML_O_2_<200 mmHg with increasing transmembrane pressure gradient or Δp (=P_pre-ML_- P_post-ML_)>30 mmHg; 2) increasing P_post-ML_CO_2_>40 mmHg and P_pre-ML_CO_2_- P_post-ML_CO_2_<10 mmHg; 3) apparent circuit thrombosis with thrombi>5 mm; 3) rising D-dimers with progressive thrombocytopenia and hyperfbrinolysis with increasing transmembrane pressure gradient and 4) unexplained haemolysis with increasing transmembrane pressure gradient [9].

### Statistical analysis

All collected data were statistically analyzed using SPSS 21.0 (Armonk, NY: IBM Corp.). Measurement data were expressed by the mean ± standard deviation (SD), and the two groups compared by analysis of variance. Count data were expressed by frequency (composition ratio), and comparison between groups was by χ^2^ test or Fisher’s exact test. *P*<0.05 indicates that the difference is statistically significant. The binary Logistic regression was conducted to analyze whether coagulation and hemolysis indicators have relationships with ICU mortality.

### Results

#### Baseline characteristics of VV ECMO patients

**Fig. 2:**
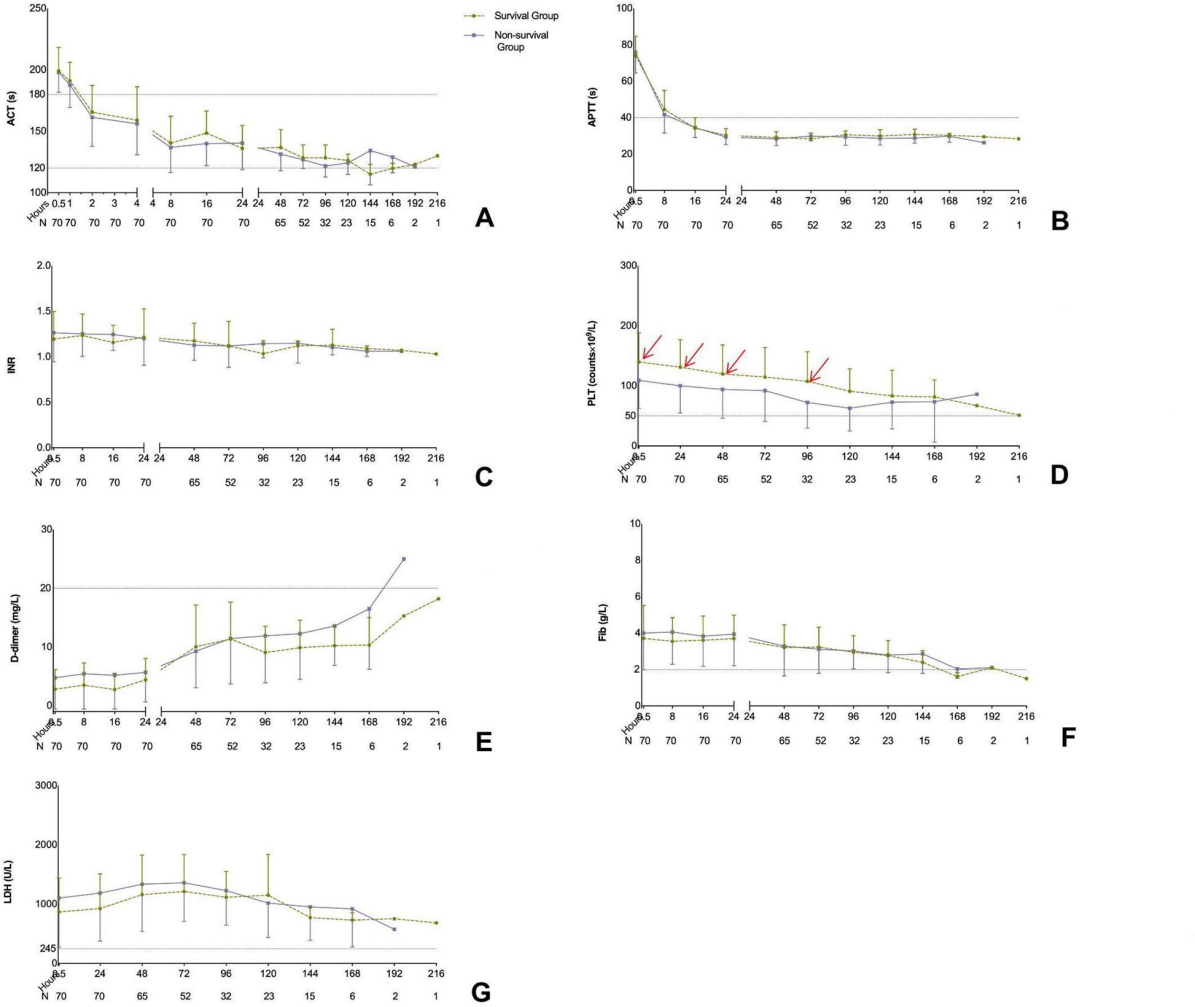
Compared of coagulation indicators in different time points between survivors and non-survivors. (**A**): ACT; (**B**): APTT; (**C**): INR; (**D**): Platelet; (**E**): D-dimer; (**F**): Fibrinogen; (**G**): LDH. The red arrows indicate statistical significance

**Fig. 3:**
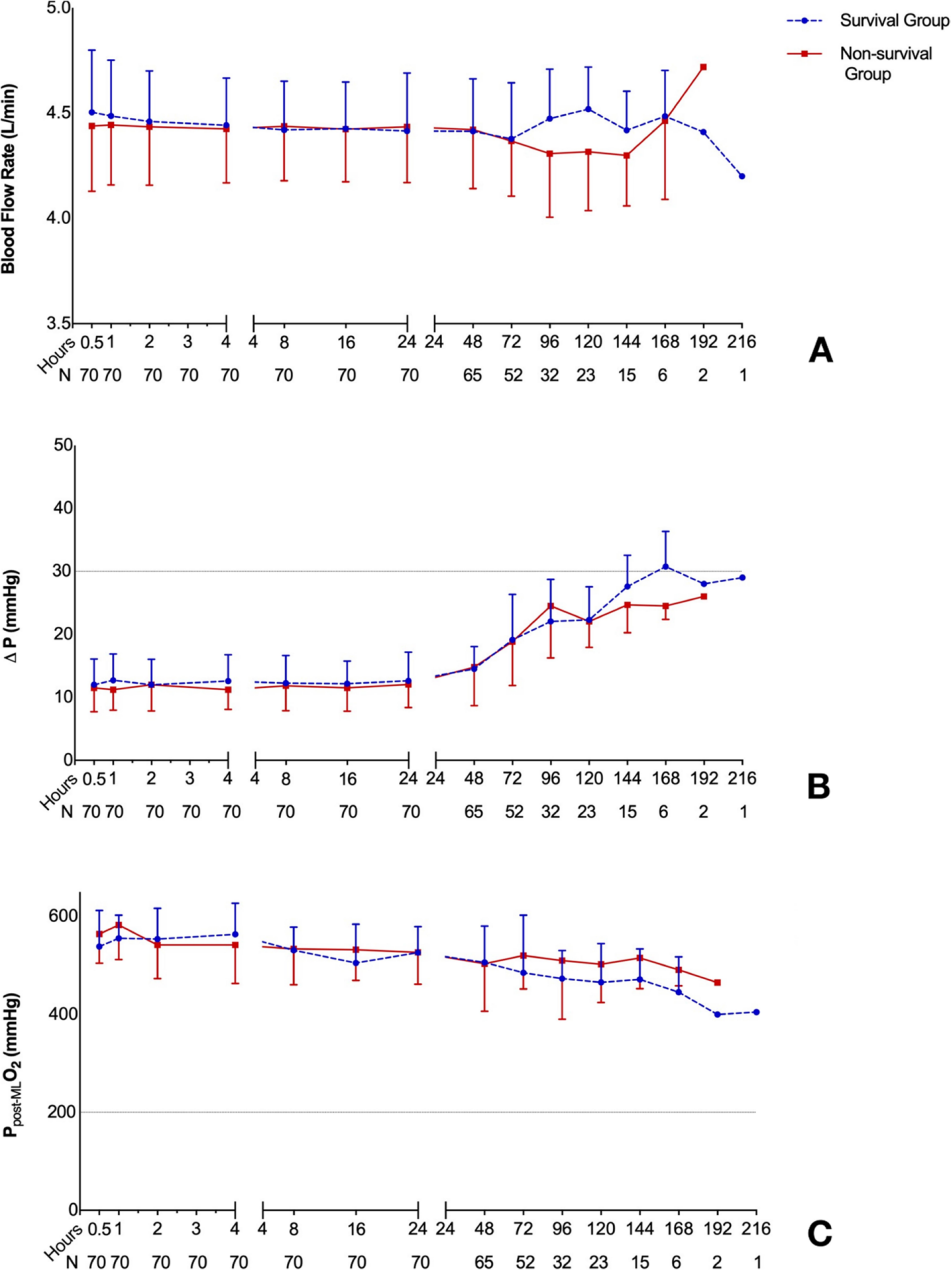
Compared of ECMO-related indicators in different time points between survivors and non-survivors. (**A**): blood flow rate; (**B**): Δp; (**C**): P_post-ML_O_2_

The characteristics of 70 enrolled patients are shown in Table 1. The longest heparin-free time was 216 h, and the mean heparin-free time was 102 h. Compared with survivors, the non-survivors were showed higher baseline SOFA score ( 8 (6.5–11) vs. 5 (4–6), *p *= 0.001, Table 1), longer time in ICU (17.16 ± 9.95d vs. 23.15 ± 10.72d, *p *= 0.022, Table 1) and lower platelet counts in 0 h, 0.5 h, 24 h, 48 h and 96 h after ECMO applied (all *p *< 0.05; Table 1, Fig. 2). However, there was no significant differences between survivors and non-survivors in ACT, APTT, INR, D-dimer, Fibrinogen, LDH, blood flow rate, Δp and P_post-ML_O_2_ of all different time point (all *p *< 0.05; Table 1, Figs. 2 and 3).

**Table 1:** Baseline characteristics of acute respiratory failure patients assisted with VV-ECMO

	**Total** (*n*=70)	**Survival Group** (*n*=25)	**Non-survival Group** (*n*=45)	***P*-**value
**Male gender**, (n%)	43 (61.4)	14 (56)	29 (64.4)	0.487
**Age** (y)	43.51 ± 13.31	40.36 ± 13.43	45.27 ± 13.07	0.141
**BMI** (Kg/M^2^)	24.49 ± 2.91	24.04 ± 2.21	24.74 ± 3.23	0.333
**SOFA score**	7 (5–9.25)	5 (4–6)	8 (6.5–11)	**0.001**
**Primary disease or comorbidities**, (n%)				0.565
Polytrauma	26 (37.1)	9 (36)	17 (37.8)	
Respiratory tract hemorrhage	10 (14.3)	3 (12)	7 (15.6)	
Gastrointestinal hemorrhage	20 (28.6)	6 (24)	14 (31.1)	
Cardiovascular surgery	8 (11.4)	3 (12)	5 (11.1)	
Non-cardiovascular surgery	6 (8.6)	4 (16)	2 (4.4)	
**Heparin free time,** (min)	102.34 ± 43.70	104.92 ± 48.01	100.91 ± 41.62	0.720
**ECMO assisted time**, (h)	190.80 ± 88.95	188.60 ± 78.34	192.02 ± 95.17	0.880
**Ventilation time**, (h)	279(214.75–432)	360(144–480)	268(220–354.5)	0.730
**tracheotomy**, (n%)	28 (40)	12 (48)	16 (35.6)	0.323
**Time in ICU**, (d)	19.30 ± 10.56	23.15 ± 10.72	17.16 ± 9.95	**0.022**
**CRRT**, (n%)	36 (51.2)	13 (52)	23 (51.1)	0.943
**PaO**_**2**_**/FiO**_**2**_	49.00 ± 7.17	54.01 ± 5.66	46.21 ± 6.41	** < 0.001**
**Baseline laboratory examinations**				
Creatine, (umol/L)	86.7 (66.95–112.75)	79 (59.5–88.5)	94(74.5–130)	**0.002**
Platelet count, (10^9^/L)	142.89 ± 49.56	163.2 ± 47.49	131.6 ± 47.50	**0.010**
Total bilirubin, (umol/L)	13.85 (8.78–25.13)	12 (8.9–22.5)	14 (8.55–27)	0.060
ACT, (s)	101.51 ± 12.76	98.64 ± 12.08	103.11 ± 12.98	0.162
APTT, (s)	34.08 ± 6.30	34.57 ± 8.13	33.8 ± 5.10	0.627
INR	1.19 ± 0.32	1.15 ± 0.32	1.20 ± 0.32	0.513
D-dimer, (mg/L)	2.65 (0.88–5.42)	1.59 (0.45–4.81)	3.18 (1.04–5.77)	0.422
Fibrinogen, (g/L)	4.09 ± 1.57	4.01 ± 1.44	4.14 ± 1.65	0.734
LDH, (U/L)	820.63 ± 527.62	751.8 ± 455.17	858.87 ± 565.12	0.420

Bold values indicate statistical significance.

*VV-ECMO* = veno-venous extracorporeal membrane oxygenation, *BMI* body mass index, *SOFA* sepsis-related organ failure assessment, *ICU* intensive care unit, *CRRT* continuous renal replacement therapy , *ACT* activated clotting time, *APTT* activated partial thromboplastin time, *INR* international normalized ratio, *LDH* lactate dehydrogenase

The data was shown as the mean ± SD, median (interquartile 25–75) or n (percentage)

Besides, the cause of death has no relationship with thrombotic disease including pulmonary embolism, acute myocardial infarction or renal thrombosis in the present study.

### Description and comparison of complications of VV ECMO

The percentage of thrombosis complications was 54.3% (38/70) including 3 oxygenator changed but there was no significant difference of complications in survival and non-survival groups ( all *p*>0.05, Table 2). In detail, the survivors were suffered 5 cases of membrane lung thrombosis, 4 cases of lower extremity venous thrombosis, 4 cases of venous cannula thrombosis, 1 case of ECMO circuit thrombosis and 1 case of oxygenator change. The non-survivors were appeared 11 cases of membrane lung thrombosis, 7 cases of lower extremity venous thrombosis, 3 cases of venous cannula thrombosis and 2 cases of oxygenator change. However, there was no bleeding complication occurred.

**Table 2:** Comparison of the complications of the survivors and non-survivors of acute respiratory failure patients assisted with VV-ECMO

	**Total** (*n *= 70)	**Survivors** (*n *= 25)	**Non-survivors** (*n *= 45)	***P*-** value
Lower extremity venous thrombosis	11 (15.7)	4 (16)	7 (15.6)	0.961
Membrane lung thrombosis	16 (22.9)	5 (20)	11 (24.4)	0.671
ECMO circuit thrombosis	1 (1.4)	1 (4)	0 (0)	0.177
Venous cannula thrombosis	7 (10)	4 (16)	3 (6.7)	0.212
Oxygenator change	3 (4.3)	1 (4)	2 (4.4)	0.930

Bold values indicate statistical significance. VV-ECMO=veno-venous extracorporeal membrane oxygenation

The data was shown as n (percentage)

### Explanations of oxygenator changed cases

The capacity of oxygen uptake was calculated as P_post_O_2_, the capacity of carbon dioxide removal was showed as P_pre_CO_2_-P_post_CO_2_, and the blood flow obstruction was described as Δp (P_pre-ML_- P_post-ML_). The oxygenator changed case 1: BFR = 3.7 L/min (BFR of the last day was 4.3 L/min at the same rotating speed), S_post_O_2_=89.2%, P_post_O_2_=61 mmHg (< 200 mmHg), P_post_CO_2_ = 49 mmHg, P_pre_CO_2_ = 40.2 mmHg, P_pre_CO_2_ -P_post_CO_2_ = 8.8 mmHg (< 10 mmHg), Δ*p*= 51 mmHg (> 30 mmHg). The oxygenator changed case 2: BFR = 3.8 L/min (BFR of the last day was 4.2 L/min at the same rotating speed), S_post_O_2_ = 98%, P_post_O_2_ = 82 mmHg (< 200 mmHg), P_post_CO_2_ = 30.7 mmHg, P_pre_CO_2_ = 36 mmHg, P_pre_CO_2_ -P_post_CO_2_ = 5.3 mmHg (< 10 mmHg), Δp = 39 mmHg (> 30 mmHg). The oxygenator changed case 3: BFR = 3.9 L/min (BFR of the last day was 4.2 L/min at the same rotating speed), S_post_O_2_ = 96.2%, P_post_O_2_ = 63 mmHg (< 200 mmHg), P_post_CO_2_ = 31 mmHg, P_pre_CO_2_ = 37 mmHg, P_pre_CO_2_ -P_post_CO_2_ = 6 mmHg (< 10 mmHg), Δp = 34 mmHg (> 30 mmHg).

### The relationship between indicators and ICU mortality

**Fig. 4:**
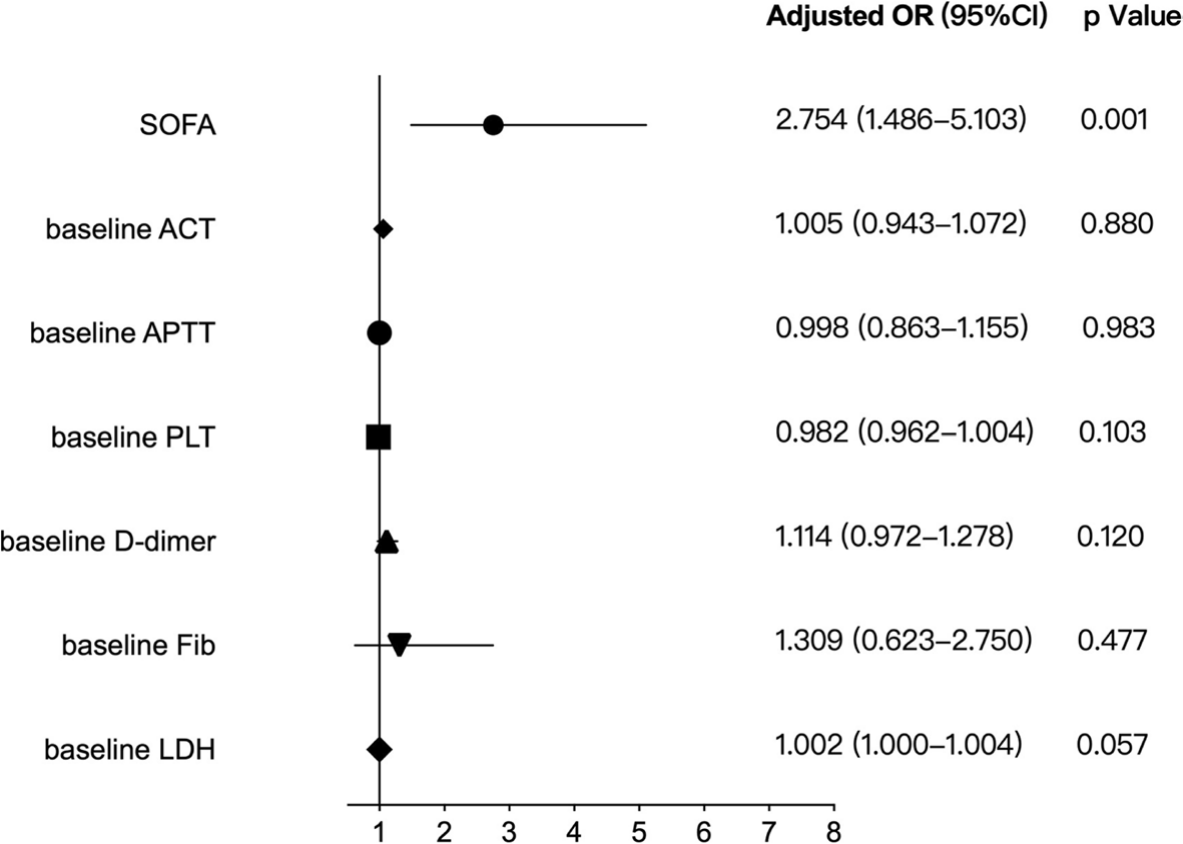
The binary Logistic regression analysis of mortality

The baseline levels of ACT, APTT, INR, platelet, D-dimer, Fibrinogen, LDH and SOFA score were choose as variables for the binary Logistic regression analysis of VV ECMO supported patients’ ICU mortality. As shown in Fig. 4, only the baseline SOFA score was significantly associated with ICU mortality (2.754 (1.486–5.103), *p *< 0.001, Fig. 4).

## Discussion

This retrospective study enrolled 70 VV ECMO patients using a new anticoagulation protocol and found no significance differences in coagulation parameters between the survivors and non-survivors, except for platelet counts at some time points and complications, showing that SOFA scores but not coagulation parameters were associated with ICU mortality and suggesting that intravenous administration of 3000 units of heparin at ECMO initiation and no continuous heparin injection in patients at risk of hemorrhage is feasible.

In recent years, due to improvements in technology and deepens in cognition gradually, ECMO has faced a dramatic growth spurt and resurgence and played an increasingly important role in saving critically ill patients. However, ECMO has been associated with a number of complications including bleeding complications, thrombo-embolic events and even end-organ dysfunction [10]. There are various anticoagulation strategy for ECMO supported patients in different centers, particularly for those receiving VV ECMO [11, 12]. The ELSO Anticoagulation Guideline recommends using systemic heparinization to run ACT of 180 and 220 s in non-bleeding patients [4]. However, the optimal anticoagulation regimen to prevent thrombosis while minimising bleeding is not known.

Many studies have been conducted on low-intensity anticoagulation during ECMO supporting. Part et al. demonstrated the safety of low dose heparin anti-coagulation regime during ECMO support for acute respiratory distress syndrome in conscious sheep [13]. A small randomized, controlled study enrolled 32 patients showed that a low heparin dose during the ECMO run was safe [14]. A number of studies have emerged in recent years which have focused on the comparison of different heparin anticoagulation regimens, often at low and standard doses [6, 7, 15]. Scholars have also conducted studies of heparin-free anticoagulation in specific patients who require ECMO support. As early as the year 2011, physicians found that the use of peripheral heparin-bonded cardiopulmonary bypass circuits without systemic heparinization reduced bleeding complications in re-operative cardiac surgery patients [16]. There have also been reports of heparin free during VA ECMO-supported lung transplantation [17]. In addition, viable case series and smaller retrospective studies using heparin-free ECMO support have been reported in postoperative cardiac surgery patients, patients with traumatic brain injury or patients with pulmonary haemorrhage [18–21]. However, those studies still do not provide evidence for an optimal anticoagulation protocol for patients undergoing ECMO. Our present study concluded that loading 3000 IU of heparin at the start of ECMO and subsequent heparin-free therapy is feasible.

However, there are fewer studies of low-dose heparin application in patients with VV ECMO, and most studies of low-dose heparin application have been in patients with VA ECMO. A recent analysis showed that a minimal heparin strategy may be protective against the major bleeding complications of VA ECMO [22]. Patients on VA ECMO often have indications for therapeutic anticoagulation due to their underlying conditions (e.g., atrial fibrillation, deep venous thrombosis, pulmonary emboli, intracardiac thrombus). Another study compared therapeutic anticoagulation (target APTT between 50 and 70 s) and low-dose heparin (aiming for APTT < 45 s) found no significant difference in the daily dose of heparin between the two groups in patients receiving VA ECMO (geometric daily mean dose of heparin 15,293 IU vs. 19,260 IU; *p *= 0.39) [23]. Our study focused on VV ECMO supported hemorrhage risk patients including polytrauma, post surgery, history of respiratory tract or gastrointestinal hemorrhage ones, and longitudinally compared the ACT, APTT, INR, D-dimer, fibrinogen, platelet, LDH, blood flow rate, Δp and P_post-ML_O_2_ of all different time point between the survivors and non-survivors. There was no bleeding complication occurred in our study, but the most common complication was thrombosis. The percentage of thrombosis complications was 54.3% (38/70) including 3 oxygenator changed.

The major problems of low-dose or no heparin anticoagulation are thromboembolic events and ECMO oxygenator change. Based on the pathophysiology of the membrane lung, ECMO oxygenator change may be required if there is an associated hematologic abnormality, an increasing obstruction to blood flow, or inadequate gas exchange [9]. Membrane lung dysfunction is associated with considerable morbidity [24]. The functions of membrane lung are carbon dioxide removal and oxygen uptake. The non-biologic surface of the membrane lung is responsible of membrane lung dysfunction, through activates inflammatory and coagulation pathways with leukocyte activation, fibrinolysis, and thrombus formation [25, 26]. Activation of coagulation and fibrinolysis might result in systemic coagulopathy or hemolysis, while clot deposition can obstruct blood flow [27, 28]. The hematologic abnormalities, mechanical obstruction, and inadequate gas exchange effects alone or together eventually lead to membrane pulmonary dysfunction [29, 30]. In our present study, there were 3 patients manifested membrane lung dysfunction and changed the ECMO oxgyenator finally because the increased obstruction to blood flow or inadequate gas exchange.

This is a retrospective study of our single-center and the sample size is small, and the future verified by multi-center, large-sample, prospective randomized controlled study is needed. This study selected VV ECMO supported acute respiratory failure patients with hemorrhage risk. For patients with no bleeding risk or severe bleeding and VA ECMO supported patients, the effect of the anticoagulant regimen is unknown. The last but not least, the indicators selected in this study are not comprehensive enough, and some new indicators, such as anti-Xa, can be added in the future.

## Conclusion

The anticoagulation protocol for hemorrhage risk patients supported by VV ECMO without heparin after a load of 3000 IU of heparin is feasible. The ACT, APTT, INR, D-dimer, fibrinogen, LDH, blood flow rate, Δp and P_post-ML_O_2_ of all different time point and the occurrences of complications have no significant difference between the survivors and non-survivors. Although the counts of platelet at some points showed significantly differences, only the SOFA score was associated with the ICU mortality in the present study. Moreover, there was no bleeding complication occurred in our study, but the most common complication was thrombosis.

## Data Availability

The datasets used and/or analysed during the current study are available from the corresponding author on reasonable request.
